# Supplementary material for: Linked within-host and between-host models and data for infectious diseases: a systematic review
Source: PeerJ. 2019 Jun 19;7:e7057. doi: 10.7717/peerj.7057 (PMC6589080; doi:10.7717/peerj.7057)
Supplement: Supplemental Information 2 [file peerj-07-7057-s002.pdf]

Linked within-host and between-host models and data for infectious diseases: A systematic review

Lauren M. Childs, Fadoua El Moustaid, Zachary Gajewski, Sarah Kadelka, Ryan Nikin-Beers, John W. Smith, Jr, Melody Walker, and Leah R. Johnson

Supplemental Figures

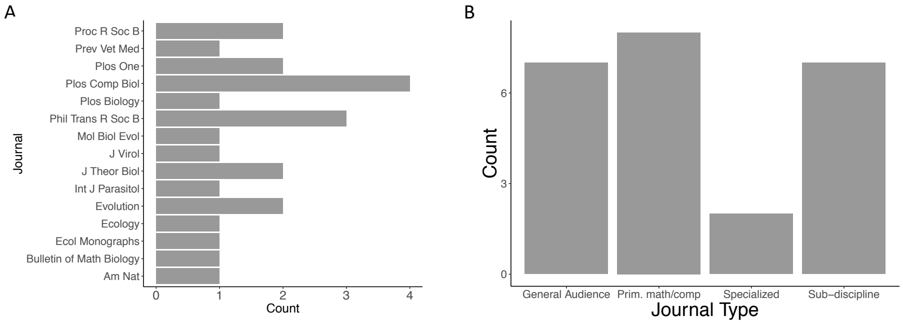

Figure S1: **Journals where included papers appeared.** (A) Counts of included papers from different journals. (B) Counts of different journal types. General audience journals included *Philosophical Transactions of the Royal Society B: Biological Sciences*, *PLOS One*, and *Proceedings of the Royal Society of London B: Biological Sciences*. Primarily mathematical and computational journals included *American Naturalist*, *Bulletin of Mathematical Biology*, *PLOS Computational Biology*, and *Journal of Theoretical Biology*. Specialized journals included *Molecular Biology and Evolution* and *Preventive Veterinary Medicine*. Sub-discipline journals included *Ecology*, *Ecological Mono Graphs*, *Evolution*, *International Journal of Parasitology*, and *Journal of Virology*. All journals of the 24 included papers appear.

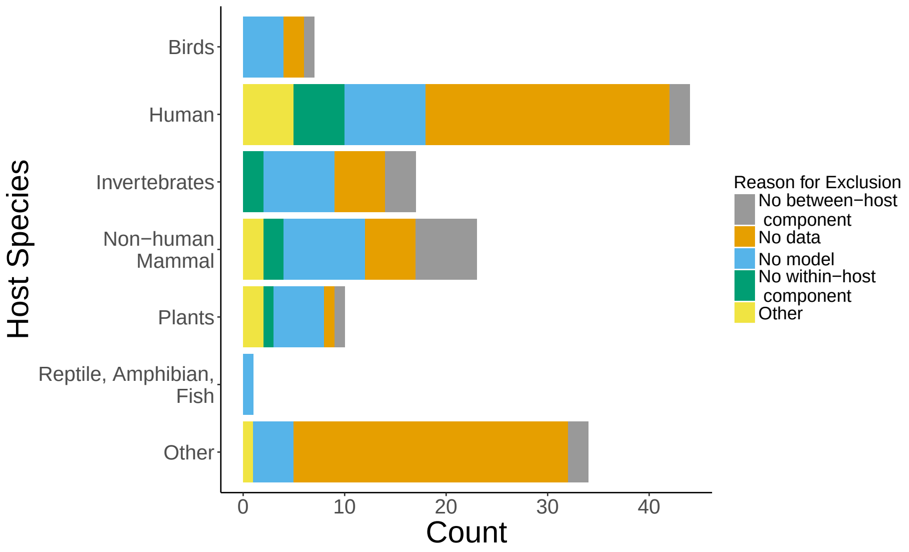

Figure S2: **Reason for exclusion of papers by host species.** The reason for exclusion was categorized as no between-host component (gray), no data (orange), no model (blue), or no within-host component (green). All other reasons were included under other (yellow). Although there may have been multiple reasons to exclude papers, only one reason was recorded.

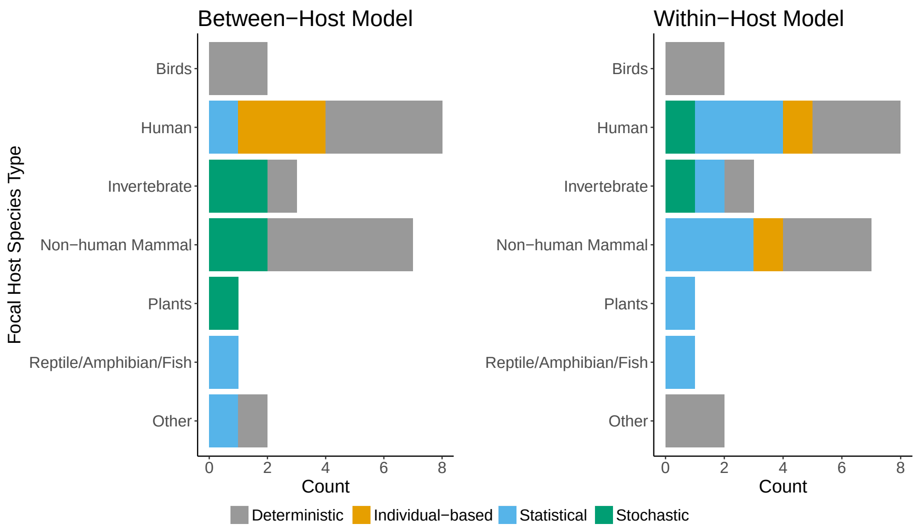

Figure S3: **Types of focal host species and the modeling type** Types of focal host species used in the within-host and between-host models and the modeling type used to represent the model components. Model types were classified as deterministic (gray), individual-based (orange), statistical (blue) or stochastic (green). The host species under Other' are bacterial species.
