# Supplementary material for: Linked within-host and between-host models and data for infectious diseases: a systematic review
Source: PeerJ. 2019 Jun 19;7:e7057. doi: 10.7717/peerj.7057 (PMC6589080; doi:10.7717/peerj.7057)
Supplement: Supplemental Information 3 — These tables summarize the answers to each of the screening or evaluation questions. [file peerj-07-7057-s003.pdf]

Supplemental Tables for: Multi-scale models and data for infectious diseases: A systematic review

S1 Exclusion Part I and Part II

Reason to exclude	Number of Papers	% of Papers
No between-host component	15	8.7
No data	64	37.0
No model	37	21.4
No within-host component	10	5.8
Other	10	5.8
Out of Scope	18	10.4
Review	19	11.0

S2 Study Properties

S2.1 Focal host species type?

Host Species	Included Papers	% of Included Papers	Excluded Papers	% of Excluded Papers
Birds	2	8.3	7	5.1
Human	8	33.3	44	32.4
Invertebrates (Insects/snails/worms/etc)	3	12.5	17	12.5
Non-human Mammal	7	29.2	23	16.9
Other	2	8.3	34	25.0
Plants	1	4.2	10	7.4
Reptile/Amphibian/Fish	1	4.2	1	0.7

S2.5 What is the type of infection?

Type of Pathogen	Number of Included Papers	Number of Excluded Papers
Bacterial	3	21
Fungal	1	5
Macroparasite (Worms/ticks/etc)	2	10
Multiple	1	2
Other	1	27
Protozoa (Malaria parasite/etc)	4	28
Viral	12	43

S4 Model Properties

S4.1 How is infection transmission modeled?

How is infection transmission modeled	Number of Papers	% of Papers
Direct contact	17	70.8
Indirect contact	5	20.8
Multiple	2	8.3

S4.2 Are the results primarily strategic or tactical?

Strategic vs Tactical Models	Number of Papers	% of Papers
Both	1	4.2
Strategic	20	83.3
Tactical	3	12.5

S4.3 What is the primary focus of the main results?

Primary Focus of Results	Number of Papers	% of Papers
Impact of between-host dynamics on within-host dynamics	2	8.3
Impact of within-host dynamics on between-host dynamics	15	62.5
Other	7	29.2

S5 Model Type

S5.1 What type of within-host model is used?

Type of Within-Model Used	Number of Papers	% of Papers
Deterministic	11	45.8
Individual-based	2	8.3
Statistical	9	37.5
Stochastic	2	8.3

S5.3 What type of between-host model is used?

Type of Between-Model Used	Number of Papers	% of Papers
Deterministic	13	54.2
Individual-based	3	12.5
Statistical	3	12.5
Stochastic	5	20.8

S5.5 How are the models linked?

Within-Between Models Linking	Number of Papers	% of Papers
Both	12	50
Within-host linked to between-host	12	50
Between-Host linked to Within-host	0	0

S5.6 Linking Mechanism, states, traits, or both?

Linking Mechanism Used	Number of Papers	% of Papers
Both	9	37.5
States	11	45.8
Traits	4	16.7

S6 Within-Host Linking Mechanisms

S6.1-5 Are the pathogen growth rate/load/death rate/immune response/symptoms used as a within-host linking mechanism?

Within-Host Linking Mechanisms	Number of Papers
Pathogen Growth Rate	5
Pathogen Load	18
Pathogen Death Rate	2
Host Immune	2
Host Symptoms	1

S7 Between-Host Linking Mechanisms

S7.1-5 Are the pathogen transmission rate/ recovery rate/ death rate/ virulence/ frequency of strains used as a between-host linking mechanism?

Between-Host Linking Mechanisms	Number of Papers
Transmission Rate	15
Host Recovery Rate	3
Host Death Rate	5
Pathogen Virulence	4
Pathogen Frequency	4

S8 Data

S8.1 Is data used at the within-host level?

Data used at Within-Host Level	Number of Papers	% of Papers
No	2	8.3
Yes	22	91.7

S8.2 If data is used at the within-host level (8.1a), is data top-down (fitting states) or bottom-up (fitting traits)?

How is data used?	Number of Papers	% of Papers
Not Data Used at Within-Host Level	2	8.3
Bottom-up	12	50.0
Top-down	10	41.7

S8.3 If data is used at the within-host level (8.1a), which fitting method is used?

Within Data Fitting Method	Number of Papers	% of Papers
Not Data Used at Within-Host Level	2	8.3
Bayesian Inference	2	8.3
Least squares	8	33.3
Maximum likelihood	7	29.2
Other	4	16.7

S8.5 Is data used for the linking mechanism?

Data used in Linking Method	Number of Papers	% of Papers
No	13	54.2
Yes	11	45.8

S8.6 If data is used for the linking mechanism (8.5a), is data top-down (fitting states) or bottom-up (fitting traits)?

How is data used?	Number of Papers	% of Papers
Not Data Used at linking Level	13	54.2
Both	1	4.2
Bottom-up	7	29.2
Top-down	3	12.5

S8.7 If data is used for the linking mechanism (8.5a), which fitting method is used?

Linking Data fitting method	Number of Papers	% of Papers
Not Data Used at linking Level	13	54.2
Least squares	4	16.7
Maximum likelihood	4	16.7
Other	3	12.5

S8.9 Is data used at the between-host level?

Data used at between-host level?	Number of Papers	% of Papers
No	15	62.5
Yes	9	37.5

S8.10 If data is used at the between-host level (8.9a), is data top-down (fitting states) or bottom-up (fitting traits)?

How is data used?	Number of Papers	% of Papers
Not Data Used at Between-Host Level	15	62.5
Both	2	8.3
Bottom-up	4	16.7
Top-down	3	12.5

S8.11 If data is used at the between-host level (8.9a), which fitting method is used?

Between-Host fitting method	Number of Papers	% of Papers
Not Data Used at Between-Host Level	16	66.7
Bayesian Inference	1	4.2
Least squares	1	4.2
Maximum likelihood	1	4.2
Other	5	20.8
