# Supplementary material for: Linked within-host and between-host models and data for infectious diseases: a systematic review
Source: PeerJ. 2019 Jun 19;7:e7057. doi: 10.7717/peerj.7057 (PMC6589080; doi:10.7717/peerj.7057)
Supplement: Supplemental Information 4 — Full screening and evaluation questions corresponding to Figure 1B in the main text. [file peerj-07-7057-s004.pdf]

1 Exclusion Part I (abstract screening)

1.1 A paper is outside the scope of our search if it is not related in any way to within-host or between-host infection dynamics. If this is the case, the paper should be rejected. Is the paper outside the scope of our search?

(a) No

(b) Yes

1.2 We also reject any review papers. Is the paper a review?

(a) No

(b) Yes

1.3 A paper should be kept if it includes a linked within-host between-host model that uses data. Otherwise, it should be rejected. Does the paper need to be further analyzed to make this determination?

(a) Yes

(b) No, it can be rejected from the abstract alone

2 Study Properties (Paper Screening)

2.1 The focal host is the main species studied. What is the focal host species type?

(a) Human

(b) Non-human mammal

(c) Reptile/Amphibian/Fish

(d) Invertebrates (Insects/snails/worms/etc)

(e) Birds

(f) Plants

(g) Other

2.2 State the focal host species scientific name.

2.3 Are there other species included in the study?

(a) Yes

(b) No

2.4 If other species are included (2.3a), state the name(s) of the other species that are included.

2.5 What is the type of infection studied?

(a) Fungal

(b) Bacterial

(c) Viral

(d) Protozoa (Malaria parasite/etc)

(e) Macroparasite (Worms/ticks/etc)

(f) Other

(g) Multiple

2.6 State the scientific name(s) of the infection(s).

2.7 If possible, state the main result of the paper.

3 Exclusion Part II (paper screening)

3.1 A paper should be kept if it includes a linked within-host between-host model that uses data. Otherwise, it should be rejected. Once the paper has been further analyzed (3.3a), should it be kept or rejected?

(a) Keep
(b) Reject

3.2 If the paper is rejected (3.3b or 3.1b), what is the main reason why it is rejected?

(a) No data
(b) No model
(c) No within-host component
(d) No between-host component
(e) Other

3.3 If the paper is rejected for another reason (3.2e), state the reason the paper should be rejected.

4 Model Properties (included papers)

4.1 How is infection transmission modeled?

(a) Direct contact
(b) Indirect contact
(c) Multiple

4.2 Are the results primarily strategic (used to understand underlying dynamics) or tactical (used to make predictions)?

(a) Strategic
(b) Tactical
(c) Both

4.3 What is the primary focus of the main result?

(a) Impact of the within-host dynamics on the between-host dynamics
(b) Impact of the between-host dynamics on the within-host dynamics
(c) Impact of the linking mechanism on the overall dynamics
(d) Other

4.4 If the main result has another focus (4.3d), state the focus of the main result.

5 Model Type (included papers)

5.1 What type of within-host model is used?

(a) Deterministic
(b) Statistical
(c) Stochastic
(d) Individual-based

(e) Other

5.2 If another type of within-host model is used (5.1e), state the type of within-host model that is used.

5.3 What type of between-host model is used?

(a) Deterministic
(b) Statistical
(c) Stochastic
(d) Individual-based
(e) Other

5.4 If another type of between-host model is used (5.3e), state the type of between-host model that is used.

5.5 How are the models linked?

(a) The within-host model is linked to the between-host model
(b) The between-host model is linked to the within-host model
(c) Both (a) and (b)
(d) Linking occurs at more than one level

5.6 Traits refer to parameters such as pathogen growth rate, while states refer to the dynamics of the system, such as pathogen load. What linking mechanisms are used?

(a) Traits
(b) States
(c) Both

6 Within-Host Linking Mechanisms (included papers)

6.1 Is the pathogen growth rate used as a within-host linking mechanism?

(a) Yes
(b) No
(c) Maybe

6.2 Is the pathogen load used as a within-host linking mechanism?

(a) Yes
(b) No
(c) Maybe

6.3 Is the pathogen death rate used as a within-host linking mechanism?

(a) Yes
(b) No
(c) Maybe

6.4 Is the immune response used as a within-host linking mechanism?

(a) Yes
(b) No

(c) Maybe

6.5 Are symptoms used as a within-host linking mechanism?

(a) Yes
(b) No
(c) Maybe

6.6 State any other within-host linking mechanisms used.

7 Between-Host Linking Mechanisms (included papers)

7.1 Is the pathogen transmission rate used as a between-host linking mechanism?

(a) Yes
(b) No
(c) Maybe

7.2 Is the host recovery rate used as a between-host linking mechanism?

(a) Yes
(b) No
(c) Maybe

7.3 Is the host death rate used as a between-host linking mechanism?

(a) Yes
(b) No
(c) Maybe

7.4 Is the pathogen virulence used as a between-host linking mechanism?

(a) Yes
(b) No
(c) Maybe

7.5 Is the frequency of strains used as a between-host linking mechanism?

(a) Yes
(b) No
(c) Maybe

7.6 State any other between-host linking mechanisms used.

8 Data (included papers)

8.1 Is data used at the within-host level?

(a) Yes
(b) No
(c) Maybe

8.2 If data is used at the within-host level (8.1a), is data top-down (fitting states) or bottom-up (fitting traits)?

(a) Top-down
(b) Bottom-up
(c) Both

8.3 If data is used at the within-host level (8.1a), which fitting method is used?

(a) Least squares
(b) Maximum likelihood
(c) Bayesian inference
(d) Other

8.4 If another fitting method is used (8.3d), state the fitting method that is used.

8.5 Is data used for the linking mechanism?

(a) Yes
(b) No
(c) Maybe

8.6 If data is used for the linking mechanism (8.5a), is data top-down (fitting states) or bottom-up (fitting traits)?

(a) Top-down
(b) Bottom-up
(c) Both

8.7 If data is used for the linking mechanism (8.5a), which fitting method is used?

(a) Least squares
(b) Maximum likelihood
(c) Bayesian inference
(d) Other

8.8 If another fitting method is used (8.7d), state the fitting method that is used.

8.9 Is data used at the between-host level?

(a) Yes
(b) No
(c) Maybe

8.10 If data is used at the between-host level (8.9a), is data top-down (fitting states) or bottom-up (fitting traits)?

(a) Top-down
(b) Bottom-up
(c) Both

8.11 If data is used at the between-host level (8.9a), which fitting method is used?

(a) Least squares
(b) Maximum likelihood
(c) Bayesian inference
(d) Other

8.12 If another fitting method is used (8.11d), state the fitting method that is used.
