# Supplementary material for: Linked within-host and between-host models and data for infectious diseases: a systematic review
Source: PeerJ. 2019 Jun 19;7:e7057. doi: 10.7717/peerj.7057 (PMC6589080; doi:10.7717/peerj.7057)
Supplement: Supplemental Information 5 — Full bibliographic information for all papers that met the criteria for inclusion in the systematic review. [file peerj-07-7057-s005.pdf]

References for Included Papers

References

[1] Benjamin M Althouse and Kathryn A Hanley. The tortoise or the hare? Impacts of within-host dynamics on transmission success of arthropod-borne viruses. *Proceedings of the Royal Society of London B: Biological Sciences*, 370(1675):20140299, 2015.

[2] Luis Fernando Chaves, Akira Kaneko, and Mercedes Pascual. Random, top-down, or bottom-up coexistence of parasites: malaria population dynamics in multi-parasitic settings. *Ecology*, 90(9):2414–2425, 2009.

[3] Shi Chen, Mike Sanderson, and Cristina Lanzas. Investigating effects of between-and within-host variability on Escherichia coli O157 shedding pattern and transmission. *Preventive Veterinary Medicine*, 109(1-2):47–57, 2013.

[4] TF Cooper and JA Heinemann. Selection for plasmid post–segregational killing depends on multiple infection: evidence for the selection of more virulent parasites through parasite–level competition. *Proceedings of the Royal Society of London B: Biological Sciences*, 272(1561):403–410, 2005.

[5] Troy Day, Samuel Alizon, and Nicole Mideo. Bridging scales in the evolution of infectious disease life histories: theory. *Evolution*, 65(12):3448–3461, 2011.

[6] John J Dennehy, Nicholas A Friedenberg, Robert D Holt, and Paul E Turner. Viral ecology and the maintenance of novel host use. *The American Naturalist*, 167(3):429–439, 2006.

[7] Greg Dwyer, Simon A Levin, and Linda Buttel. A simulation model of the population dynamics and evolution of myxomatosis. *Ecological Monographs*, 60(4):423–447, 1990.

[8] Helen R Fryer, John Frater, Anna Duda, Duncan Palmer, Rodney E Phillips, and Angela R McLean. Cytotoxic T-lymphocyte escape mutations identified by HLA association favor those which escape and revert rapidly. *Journal of Virology*, 86(16):8568–8580, 2012.

[9] Federica Giardina, Ethan Obie Romero-Severson, Jan Albert, Tom Britton, and Thomas Leitner. Inference of transmission network structure from HIV phylogenetic trees. *PLoS Computational Biology*, 13(1):e1005316, 2017.

[10] Matthew D Hall and Nicole Mideo. Linking sex differences to the evolution of infectious disease life-histories. *Philosophical Transactions of the Royal Society B: Biological Sciences*, 373(1757):20170431, 2018.

[11] Andreas Handel, Justin Brown, David Stallknecht, and Pejman Rohani. A multi-scale analysis of influenza A virus fitness trade-offs due to temperature-dependent virus persistence. *PLoS Computational Biology*, 9(3):e1002989, 2013.

[12] Andreas Handel, Camille Lebarbenchon, David Stallknecht, and Pejman Rohani. Trade-offs between and within scales: environmental persistence and within-host fitness of avian influenza viruses. *Proceedings of the Royal Society of London B: Biological Sciences*, 281(1787):20133051, 2014.

[13] David Kennedy and Greg Dwyer. Effects of multiple sources of genetic drift on pathogen variation within hosts. *PLoS Biology*, 16(3):e2004444, 2018.

[14] Melen Leclerc, Thierry Doré, Christopher A Gilligan, Philippe Lucas, and João AN Filipe. Estimating the delay between host infection and disease (incubation period) and assessing its significance to the epidemiology of plant diseases. *PLoS One*, 9(1):e86568, 2014.

[15] Heather M Lindberg, Kurt A McKean, T Caraco, and Nang Wang. Within-host dynamics and random duration of pathogen infection: Implications for between-host transmission. *Journal of Theoretical Biology*, 446:137–148, 2018.

[16] F Ellis McKenzie and William H Bossert. An integrated model of *Plasmodium falciparum* dynamics. *Journal of Theoretical Biology*, 232(3):411–426, 2005.

[17] Nicole Mideo, William A Nelson, Sarah E Reece, Andrew S Bell, Andrew F Read, and Troy Day. Bridging scales in the evolution of infectious disease life histories: application. *Evolution*, 65(11):3298–3310, 2011.

[18] Leslie A Reperant, Thijs Kuiken, Bryan T Grenfell, Albert DME Osterhaus, and Andrew P Dobson. Linking influenza virus tissue tropism to population-level reproductive fitness. *PLoS One*, 7(8):e43115, 2012.

[19] Jessica F Stephenson, Kyle A Young, Jordan Fox, Jukka Jokela, Joanne Cable, and Sarah E Perkins. Host heterogeneity affects both parasite transmission to and fitness on subsequent hosts. *Philosophical Transaction of the Royal Society of London B: Biological Sciences*, 372(1719):20160093, 2017.

[20] Katsuhisa Takumi, Frits Franssen, Manoj Fonville, Aurélie Grasset, Isabelle Vallée, Pascal Boireau, Peter Teunis, and Joke van der Giessen. Within-host dynamics of *Trichinella spiralis* predict persistent parasite transmission in rat populations. *International Journal for Parasitology*, 40:1317–1324, 2010.

[21] Necibe Tuncer, Hayriye Gulbudak, Vincent L Cannataro, and Maia Martcheva. Structural and practical identifiability issues of immuno-epidemiological vector–host models with application to rift valley fever. *Bulletin of Mathematical Biology*, 78:1796–1827, 2016.

[22] Christiaan H van Dorp, Michiel van Boven, and Rob J De Boer. Immuno-epidemiological modeling of HIV-1 predicts high heritability of the set-point virus load, while selection for CTL escape dominates virulence evolution. *PLoS Computational Biology*, 10(12):e1003899, 2014.

[23] Erik M Volz, Ethan Romero-Severson, and Thomas Leitner. Phylodynamic inference across epidemic scales. *Molecular Biology and Evolution*, 34(5):1276–1288, 2017.

[24] Bram Vrancken, Andrew Rambaut, Marc A Suchard, Alexei Drummond, Guy Baele, Inge Derdelinckx, Eric Van Wijngaerden, Anne-Mieke Vandamme, Kristel Van Laethem, and Philippe Lemey. The genealogical population dynamics of HIV-1 in a large transmission chain: bridging within and among host evolutionary rates. *PLoS Computational Biology*, 10(4):e1003505, 2014.
