# Supplementary material for: Linked within-host and between-host models and data for infectious diseases: a systematic review
Source: PeerJ. 2019 Jun 19;7:e7057. doi: 10.7717/peerj.7057 (PMC6589080; doi:10.7717/peerj.7057)
Supplement: Supplemental Information 6 — Full bibliographic information for all papers that did not meet the criteria for inclusion in the systematic review, and so were excluded. [file peerj-07-7057-s006.pdf]

References for Excluded Papers

References

[1] Murray E Alexander, Sarah M Dietrich, Yi Hua, and Seyed M Moghadas. A comparative evaluation of modelling strategies for the effect of treatment and host interactions on the spread of drug resistance. *Journal of Theoretical Biology*, 259(2):253–263, 2009.

[2] Samuel Alizon and Christophe Fraser. Within-host and between-host evolutionary rates across the HIV-1 genome. *Retrovirology*, 10(1):49, 2013.

[3] Samuel Alizon and Sébastien Lion. Within-host parasite cooperation and the evolution of virulence. *Proceedings of the Royal Society of London B: Biological Sciences*, 278:3738–3747, 2011.

[4] Samuel Alizon, Fabio Luciani, and Roland R Regoes. Epidemiological and clinical consequences of within-host evolution. *Trends in Microbiology*, 19(1):24–32, 2011.

[5] Samuel Alizon and Minus Van Baalen. Acute or chronic? Within-host models with immune dynamics, infection outcome, and parasite evolution. *The American Naturalist*, 172(6):E244–E256, 2008.

[6] Michael F Antolin. Unpacking β: within-host dynamics and the evolutionary ecology of pathogen transmission. *Annual Review of Ecology, Evolution, and Systematics*, 39:415–437, 2008.

[7] Yael Artzy-Randrup and Mercedes Pascual. Composite temporal strategies in pathogen evolution: balancing invasion and persistence. *Theoretical Ecology*, 7(4):325–334, 2014.

[8] Ben Ashby and Kayla C King. Friendly foes: the evolution of host protection by a parasite. *Evolution Letters*, 1(4):211–221, 2017.

[9] Stephanie Bedhomme, Philip Agnew, Christine Sidobre, and Yannis Michalakis. Virulence reaction norms across a food gradient. *Proceedings of the Royal Society of London B: Biological Sciences*, 271(1540):739, 2004.

[10] Clayton C Beegle and Earl R Oatman. Effect of a nuclear polyhedrosis virus on the relationship between *Trichoplusia ni (Lepidoptera: Noctuidae)* and the parasite, *Hyposoter exiguae (Hymenoptera: Ichneumonidae)*. *Journal of Invertebrate Pathology*, 25(1):59–71, 1975.

[11] Graham Bell and Craig MacLean. The search for 'evolution-proof' antibiotics. *Trends in Microbiology*, 26(6):471–483, 2018.

[12] Mónica Betancourt, Fernando Escriu, Aurora Fraile, and Fernando García-Arenal. Virulence evolution of a generalist plant virus in a heterogeneous host system. *Evolutionary Applications*, 6(6):875–890, 2013.

[13] Mónica Betancourt, Aurora Fraile, and Fernando García-Arenal. Cucumber mosaic virus satellite RNAs that induce similar symptoms in melon plants show large differences in fitness. *Journal of General Virology*, 92(8):1930–1938, 2011.

[14] Philip LG Birget, Charlotte Repton, Aidan J O'Donnell, Petra Schneider, and Sarah E Reece. Phenotypic plasticity in reproductive effort: malaria parasites respond to resource availability. *Proceedings of the Royal Society of London B: Biological Sciences*, 284(1860):20171229, 2017.

[15] Barbara Boldin and Odo Diekmann. Superinfections can induce evolutionarily stable coexistence of pathogens. *Journal of Mathematical Biology*, 56(5):635–672, 2008.

[16] John D Boone, Kenneth C McGwire, Elmer W Otteson, Robert S DeBaca, Edward A Kuhn, and Stephen C St Jeor. Infection dynamics of Sin Nombre virus after a widespread decline in host populations. *The American Journal of Tropical Medicine and Hygiene*, 67(3):310–318, 2002.

[17] Christine Braquart-Varnier, Maryline Raimond, Gaëtan Mappa, Frédéric D Chevalier, Le Clech, Mathieu Sicard, et al. The hematopoietic organ: a cornerstone for *Wolbachia* propagation between and within hosts. *Frontiers in Microbiology*, 6:1424, 2015.

[18] Ellen Brooks-Pollock and James LN Wood. Eliminating bovine tuberculosis in cattle and badgers: insight from a dynamic model. *Proceedings of the Royal Society of London B: Biological Sciences*, 282(1808):20150374, 2015.

[19] Mary Bushman, Rustom Antia, Venkatachalam Udhayakumar, and Jacobus C de Roode. Within-host competition can delay evolution of drug resistance in malaria. *PLoS Biology*, 16(8):e2005712, 2018.

[20] Liming Cai, Necibe Tuncer, and Maia Martcheva. How does within-host dynamics affect population-level dynamics? Insights from an immuno-epidemiological model of malaria. *Mathematical Methods in the Applied Sciences*, 40(18):6424–6450, 2017.

[21] Xiuli Cen, Zhilan Feng, and Yulin Zhao. Emerging disease dynamics in a model coupling within-host and between-host systems. *Journal of Theoretical Biology*, 361:141–151, 2014.

[22] Hsiao-Han Chang and Daniel L Hartl. Recurrent bottlenecks in the malaria life cycle obscure signals of positive selection. *Parasitology*, 142:S98–S107, 2015.

[23] Daniel Coombs, Michael A Gilchrist, and Colleen L Ball. Evaluating the importance of within-and between-host selection pressures on the evolution of chronic pathogens. *Theoretical Population Biology*, 72(4):576–591, 2007.

[24] Diego F Cuadros and Gisela García-Ramos. Variable effect of co-infection on the HIV infectivity: within-host dynamics and epidemiological significance. *Theoretical Biology and Medical Modelling*, 9(1):9, 2012.

[25] Yan-Xia Dang, Xue-Zhi Li, and Maia Martcheva. Competitive exclusion in a multi-strain immuno-epidemiological influenza model with environmental transmission. *Journal of Biological Dynamics*, 10(1):416–456, 2016.

[26] Nicola De Maio, Chieh-Hsi Wu, and Daniel J Wilson. SCOTTI: efficient reconstruction of transmission within outbreaks with the structured coalescent. *PLoS Computational Biology*, 12(9):e1005130, 2016.

[27] Stacy L DeBlasio, Juan D Chavez, Mariko M Alexander, John Ramsey, Jimmy K Eng, Jaclyn Mahoney, Stewart M Gray, James E Bruce, and Michelle Cilia. Visualization of host-polerovirus interaction topologies using protein interaction reporter technology. *Journal of Virology*, 90(4):1973–1987, 2016.

[28] Christian A Devaux. Emerging and re-emerging viruses: A global challenge illustrated by Chikungunya virus outbreaks. *World Journal of Virology*, 1(1):11, 2012.

[29] Caroline E Dewar, Paula MacGregor, Sinclair Cooper, Matthew K Gould, Keith R Matthews, Nicholas J Savill, and Achim Schnaufer. Mitochondrial DNA is critical for longevity and metabolism of transmission stage *Trypanosoma brucei*. *PLoS Pathogens*, 14(7):e1007195, 2018.

[30] Hilje M Doekes, Christophe Fraser, and Katrina A Lythgoe. Effect of the latent reservoir on the evolution of HIV at the within-and between-host levels. *PLoS Computational Biology*, 13(1):e1005228, 2017.

[31] Patrick T Dolan, Zachary J Whitfield, and Raul Andino. Mechanisms and concepts in RNA virus population dynamics and evolution. *Annual Review of Virology*, 5(1):69–92, 2018.

[32] Narmugesalsadat Dorratoltaj, Ryan Nikin-Beers, Stanca M Ciupe, Stephen G Eubank, and Kaja M Abbas. Multi-scale immunoepidemiological modeling of within-host and between-host HIV dynamics: systematic review of mathematical models. *PeerJ*, 5:e3877, 2017.

[33] Alison B Duncan, Eike Dusi, Martina Schrallhammer, Thomas Berendonk, and Oliver Kaltz. Population-level dynamics in experimental mixed infections: evidence for competitive exclusion among bacterial parasites of *Paramecium caudatum*. *Oikos*, 127:1380–1389, 2018.

[34] Stephen P Ellner, Laura E Jones, Laura D Mydlarz, and C Drew Harvell. Within-host disease ecology in the sea fan *Gorgonia ventalina*: modeling the spatial immunodynamics of a coral-pathogen interaction. *The American Naturalist*, 170(6):E143–E161, 2007.

[35] Silvio Erler, Mario Popp, Stephan Wolf, and H Michael G Lattorff. Sex, horizontal transmission, and multiple hosts prevent local adaptation of *Crithidia bombi*, a parasite of bumblebees (*Bombus spp.*). *Ecology and Evolution*, 2(5):930–940, 2012.

[36] Michael Famulare, Christian Selinger, Kevin A McCarthy, Philip A Eckhoff, and Guillaume Chabot-Couture. Assessing the stability of polio eradication after the withdrawal of oral polio vaccine. *PLoS Biology*, 16(4):e2002468, 2018.

[37] Rubaiyea Farrukee, Zarbreski E Alex, McCaw James, Jesse D Bloom, Patrick Reading, and Aeron Christopher Hurt. Characterization of influenza B virus variants with reduced neuraminidase inhibitor susceptibility. *Antimicrobial Agents and Chemotherapy*, 62(11):e01081–18, 2018.

[38] Zhilan Feng, Xiuli Cen, Yulin Zhao, and Jorge X Velasco-Hernandez. Coupled within-host and between-host dynamics and evolution of virulence. *Mathematical Biosciences*, 270:204–212, 2015.

[39] Zhilan Feng, Jorge Velasco-Hernandez, and Brenda Tapia-Santos. A mathematical model for coupling within-host and between-host dynamics in an environmentally-driven infectious disease. *Mathematical Biosciences*, 241(1):49–55, 2013.

[40] Andy Fenton. Editorial: Mathematical modelling of infectious diseases. *Parasitology*, 143(7):801–804, 2016.

[41] Sofía Fernández-González, Antón Pérez-Rodríguez, Heather C Proctor, Iván De la Hera, and Javier Pérez-Tris. High diversity and low genetic structure of feather mites associated with a phenotypically variable bird host. *Parasitology*, 145:1–8, 2018.

[42] Naomi L Forrester, Serafín Gutiérrez, and Lark L Coffey. The evolution and transmission of vector-borne viruses. *Virus Evolution*, page 61, 2016.

[43] Simon DW Frost, Oliver G Pybus, Julia R Gog, Cecile Viboud, Sebastian Bonhoeffer, and Trevor Bedford. Eight challenges in phylodynamic inference. *Epidemics*, 10:88–92, 2015.

[44] Helen R Fryer and Angela R McLean. Modelling the spread of HIV immune escape mutants in a vaccinated population. *PLoS Computational Biology*, 7(12):e1002289, 2011.

[45] Helen R Fryer and Angela R McLean. There is no safe dose of prions. *PLoS One*, 6(8):e23664, 2011.

[46] Vitaly V Ganusov, Carl T Bergstrom, and Rustom Antia. Within-host population dynamics and the evolution of microparasites in a heterogeneous host population. *Evolution*, 56(2):213–223, 2002.

[47] László Zsolt Garamszegi. Patterns of co-speciation and host switching in primate malaria parasites. *Malaria Journal*, 8(1):110, 2009.

[48] Winston Garira, Dephney Mathebula, and Rendani Netshikweta. A mathematical modelling framework for linked within-host and between-host dynamics for infections with free-living pathogens in the environment. *Mathematical Biosciences*, 256:58–78, 2014.

[49] Michael A Gilchrist and Daniel Coombs. Evolution of virulence: interdependence, constraints, and selection using nested models. *Theoretical Population Biology*, 69(2):145–153, 2006.

[50] Michael A Gilchrist and Akira Sasaki. Modeling host–parasite coevolution: a nested approach based on mechanistic models. *Journal of Theoretical Biology*, 218(3):289–308, 2002.

[51] Erida Gjini, Daniel T Haydon, JD Barry, and Christina A Cobbold. Linking the antigen archive structure to pathogen fitness in African trypanosomes. *Proceedings of the Royal Society of London B: Biological Sciences*, 280(1753):20122129, 2013.

[52] Isabel Gordo and Paulo RA Campos. Patterns of genetic variation in populations of infectious agents. *BMC Evolutionary Biology*, 7(1):116, 2007.

[53] Andrea L Graham, Isabella M Cattadori, James O Lloyd-Smith, Matthew J Ferrari, and Ottar N Bjørnstad. Transmission consequences of coinfection: cytokines writ large? *Trends in Parasitology*, 23(6):284–291, 2007.

[54] Jaco M Greeff, Kerry Reid, Janishtha R Gagjee, Sarah J Clift, and Pamela J de Waal. Population genetic structure of the parasitic nematode *Spirocerca lupi* in south africa. *Veterinary Parasitology*, 258:64–69, 2018.

[55] Philip B Greenspoon, Sydney Banton, and Nicole Mideo. Immune system handling time may alter the outcome of competition between pathogens and the immune system. *Journal of Theoretical Biology*, 447:25–31, 2018.

[56] Hayriye Gulbudak, Vincent L Cannataro, Necibe Tuncer, and Maia Martcheva. Vector-borne pathogen and host evolution in a structured immuno-epidemiological system. *Bulletin of Mathematical Biology*, 79(2):325–355, 2017.

[57] Serafín Gutiérrez, Gaël Thébaud, Darci R Smith, Joan L Kenney, and Scott C Weaver. Demographics of natural oral infection of mosquitos by Venezuelan equine encephalitis virus. *Journal of Virology*, 89(7):4020–4022, 2015.

[58] Gerod S Hall and Damon P Little. Within-host competition between barley yellow dwarf-PAV and-PAS. *Virus Research*, 174(1-2):148–151, 2013.

[59] Spencer R Hall, Claes R Becker, Meghan A Duffy, and Carla E Cáceres. A power-efficiency trade-off in resource use alters epidemiological relationships. *Ecology*, 93(3):645–656, 2012.

[60] Andreas Handel and Pejman Rohani. Crossing the scale from within-host infection dynamics to between-host transmission fitness: a discussion of current assumptions and knowledge. *Philosophical Transactions of the Royal Society B: Biological Sciences*, 370(1675):20140302, 2015.

[61] Dana M Hawley and Sonia M Altizer. Disease ecology meets ecological immunology: understanding the links between organismal immunity and infection dynamics in natural populations. *Functional Ecology*, 25(1):48–60, 2011.

[62] Barbara Hellriegel. Immunoepidemiology–bridging the gap between immunology and epidemiology. *Trends in Parasitology*, 17(2):102–106, 2001.

[63] Jessica L Hite and Clayton E Cressler. Resource-driven changes to host population stability alter the evolution of virulence and transmission. *Philosophical Transactions of the Royal Society B: Biological Sciences*, 373:20170087, 2018.

[64] Thomas Holding, John Joseph Valletta, and Mario Recker. Multiscale immune selection and the transmission-diversity feedback in antigenically diverse pathogen systems. *The American Naturalist*, 192(6):E189–E201, 2018.

[65] Robert D Holt and John Pickering. Infectious disease and species coexistence: a model of Lotka-Volterra form. *The American Naturalist*, 126(2):196–211, 1985.

[66] Anna Hool, Gabriel E Leventhal, and Sebastian Bonhoeffer. Virus-induced target cell activation reconciles set-point viral load heritability and within-host evolution. *Epidemics*, 5(4):174–180, 2013.

[67] Aeron C Hurt, Siti Sarah Nor'e, James M McCaw, Helen R Fryer, Jennifer Mosse, Angela R McLean, and Ian G Barr. Assessing the viral fitness of *oseltamivir*-resistant influenza viruses in ferrets, using a competitive-mixtures model. *Journal of Virology*, 84(18):9427–9438, 2010.

[68] Paul J Hurtado. Within-host dynamics of mycoplasma infections: Conjunctivitis in wild passerine birds. *Journal of Theoretical Biology*, 306:73–92, 2012.

[69] Mark W Jackwood, David Hall, and Andreas Handel. Molecular evolution and emergence of avian gammacoronaviruses. *Infection, Genetics and Evolution*, 12(6):1305–1311, 2012.

[70] John Jaenike. Coupled population dynamics of endosymbionts within and between hosts. *Oikos*, 118(3):353–362, 2009.

[71] Pieter TJ Johnson and Ian D Buller. Parasite competition hidden by correlated coinfection: using surveys and experiments to understand parasite interactions. *Ecology*, 92(3):535–541, 2011.

[72] Rowland R Kao. Networks and models with heterogeneous population structure in epidemiology. In *Network Science*, pages 51–84. Springer, 2010.

[73] A Karvonen, G-H Cheng, O Seppälä, and ET Valtonen. Intestinal distribution and fecundity of two species of *Diplostomum* parasites in definitive hosts. *Parasitology*, 132(3):357–362, 2006.

[74] Elisabeth Kernbauer, Katie Maurer, Victor J Torres, Bo Shopsin, and Ken Cadwell. Gastrointestinal dissemination and transmission of *Staphylococcus aureus* following bacteremia. *Infection and Immunity*, 83(1):372–378, 2015.

[75] Aaron A King, Sourya Shrestha, Eric T Harvill, and Ottar N Bjørnstad. Evolution of acute infections and the invasion-persistence trade-off. *The American Naturalist*, 173(4):446–455, 2009.

[76] Eili Y Klein. The impact of heterogeneous transmission on the establishment and spread of antimalarial drug resistance. *Journal of Theoretical Biology*, 340:177–185, 2014.

[77] D Klinkenberg and JAP Heesterbeek. A simple model for the within-host dynamics of a protozoan parasite. *Proceedings of the Royal Society of London B: Biological Sciences*, 272(1563):593–600, 2005.

[78] Maisem Laabei, Anne-Catrin Uhlemann, Franklin D Lowy, Eloise D Austin, Maho Yokoyama, Khadija Ouadi, Edward Feil, Harry A Thorpe, Barnabas Williams, Mark Perkins, et al. Evolutionary trade-offs underlie the multi-faceted virulence of *Staphylococcus aureus*. *PLoS Biology*, 13(9):e1002229, 2015.

[79] Louis Lambrechts and Sebastian Lequime. Evolutionary dynamics of dengue virus populations within the mosquito vector. *Current Opinion in Virology*, 21:47–53, 2016.

[80] Alexander Lange and Neil M Ferguson. Antigenic diversity, transmission mechanisms, and the evolution of pathogens. *PLoS Computational Biology*, 5(10):e1000536, 2009.

[81] Jennie S Lavine, Mary Poss, and Bryan T Grenfell. Directly transmitted viral diseases: modeling the dynamics of transmission. *Trends in Microbiology*, 16(4):165–172, 2008.

[82] Mathieu Legros and Sebastian Bonhoeffer. A combined within-host and between-hosts modelling framework for the evolution of resistance to antimalarial drugs. *Journal of the Royal Society Interface*, 13(117):20160148, 2016.

[83] You-Yu Lin, Chieh Liu, Wei-Hung Chien, Li-Ling Wu, Yong Tao, Dafei Wu, Xuemei Lu, Chia-Hung Hsieh, Pei-Jer Chen, Hurng-Yi Wang, et al. New insights into the evolutionary rate of hepatitis B virus at different biological scales. *Journal of Virology*, 89(7):3512–3522, 2015.

[84] Sébastien Lion. Multiple infections, kin selection and the evolutionary epidemiology of parasite traits. *Journal of Evolutionary Biology*, 26(10):2107–2122, 2013.

[85] Fabio Luciani and Samuel Alizon. The evolutionary dynamics of a rapidly mutating virus within and between hosts: the case of hepatitis C virus. *PLoS Computational Biology*, 5(11):e1000565, 2009.

[86] Casper K Lumby, Nuno R Nene, and Christopher JR Illingworth. A novel framework for inferring parameters of transmission from viral sequence data. *PLoS Genetics*, 14(10):e1007718, 2018.

[87] Katrina A Lythgoe, Lorenzo Pellis, and Christophe Fraser. Is HIV short-sighted? Insights from a multistrain nested model. *Evolution*, 67(10):2769–2782, 2013.

[88] MJ Mackinnon and A Read. Selection for high and low virulence in the malaria parasite. *Proceedings of the Royal Society of London B: Biological Sciences*, 266(1420):741–748, 1999.

[89] Sean P Maher and Robert M Timm. Patterns of host and flea communities along an elevational gradient in Colorado. *Canadian Journal of Zoology*, 92(5):433–442, 2014.

[90] Katherine M Marchetto and Alison G Power. Context-dependent interactions between pathogens and a mutualist affect pathogen fitness and mutualist benefits to hosts. *Ecology*, 99(12):2833–2843, 2018.

[91] Maia Martcheva, Suzanne Lenhart, Shigetoshi Eda, Don Klinkenberg, Eiichi Momotani, and Judy Stabel. An immuno-epidemiological model for Johnes disease in cattle. *Veterinary Research*, 46(1):69, 2015.

[92] Maia Martcheva and Xue-Zhi Li. Linking immunological and epidemiological dynamics of HIV: the case of super-infection. *Journal of Biological Dynamics*, 7(1):161–182, 2013.

[93] Micaela Martinez-Bakker and Barbara Helm. The influence of biological rhythms on host-parasite interactions. *Trends in Ecology & Evolution*, 30(6):314–326, 2015.

[94] Helen O McCarthy, Susan Fitzpatrick, and S W B Irwin. Life history and life cycles: production and behavior of trematode cercariae in relation to host exploitation and next-host characteristics. *Journal of Parasitology*, 88(5):910–918, 2002.

[95] Alexa Fritzsche McKay and Bethany J Hoye. Are migratory animals superspreaders of infection? *Integrative and Comparative Biology*, 56(2):260–267, 2016.

[96] Nicole Mideo, Alvaro Acosta-Serrano, Toni Aebischer, Mark JF Brown, Andy Fenton, Ville-Petri Friman, Olivier Restif, Sarah E Reece, Joanne P Webster, and Sam P Brown. Life in cells, hosts, and vectors: parasite evolution across scales. *Infection, Genetics and Evolution*, 13:344–347, 2013.

[97] Nicole Mideo, Samuel Alizon, and Troy Day. Linking within-and between-host dynamics in the evolutionary epidemiology of infectious diseases. *Trends in Ecology & Evolution*, 23(9):511–517, 2008.

[98] Nicole Mideo, Nicholas J Savill, William Chadwick, Petra Schneider, Andrew F Read, Troy Day, and Sarah E Reece. Causes of variation in malaria infection dynamics: insights from theory and data. *The American Naturalist*, 178(6):174–188, 2011.

[99] Victor A Mobegi, Craig W Duffy, Alfred Amambua-Ngwa, Kovana M Loua, Eugene Laman, Davis C Nwakanma, Bronwyn MacInnis, Harvey Aspeling-Jones, Lee Murray, Taane G Clark, et al. Genome-wide analysis of selection on the malaria parasite *Plasmodium falciparum* in West African populations of differing infection endemicity. *Molecular Biology and Evolution*, 31(6):1490–1499, 2014.

[100] Louise H Moncla, Gongxun Zhong, Chase W Nelson, Jorge M Dinis, James Mutschler, Austin L Hughes, Tokiko Watanabe, Yoshihiro Kawaoka, and Thomas C Friedrich. Selective bottlenecks shape evolutionary pathways taken during mammalian adaptation of a 1918-like avian influenza virus. *Cell Host & Microbe*, 19(2):169–180, 2016.

[101] Erin A Mordecai, Madeleine Hindenlang, and Charles E Mitchell. Differential impacts of virus diversity on biomass production of a native and an exotic grass host. *PLoS One*, 10(7):e0134355, 2015.

[102] Richard Moxon and Edo Kussell. The impact of bottlenecks on microbial survival, adaptation, and phenotypic switching in host-pathogen interactions. *Evolution*, 71(12):2803–2816, 2017.

[103] Carmen Lía Murall, Chris T Bauch, and Troy Day. Could the human papillomavirus vaccines drive virulence evolution? *Proceedings of the Royal Society of London B: Biological Sciences*, 282(1798):20141069, 2015.

[104] Lisa N Murillo, Michael S Murillo, and Alan S Perelson. Towards multiscale modeling of influenza infection. *Journal of Theoretical Biology*, 332:267–290, 2013.

[105] Rendani Netshikweta and Winston Garira. A multiscale model for the world's first parasitic disease targeted for eradication: guinea worm disease. *Computational and Mathematical Methods in Medicine*, 2017, 2017.

[106] Aidan J O'Donnell, Petra Schneider, Harriet G McWatters, and Sarah E Reece. Fitness costs of disrupting circadian rhythms in malaria parasites. *Proceedings of the Royal Society of London B: Biological Sciences*, 278:2429–2436, 2011.

[107] ZB Osiemo, A Marten, M Kaib, LM Gitonga, HI Boga, and R Brandl. Open relationships in the castles of clay: high diversity and low host specificity of *Termitomyces fungi* associated with fungus-growing termites in Africa. *Insectes Sociaux*, 57(3):351–363, 2010.

[108] Israel Pagán, Carlos Alonso-Blanco, and Fernando García-Arenal. The relationship of within-host multiplication and virulence in a plant-virus system. *PLoS One*, 2(8):e786, 2007.

[109] Israel Pagán, Patricia Rojas, José Tomás Ramos, and África Holguín. Clinical determinants of HIV-1B between-host evolution and their association with drug resistance in pediatric patients. *PLoS One*, 11(12):e0167383, 2016.

[110] Evan C Palmer-Young, Austin C Calhoun, Anastasiya Mirzayeva, and Ben M Sadd. Effects of the floral phytochemical eugenol on parasite evolution and bumble bee infection and preference. *Scientific Reports*, 8:2074, 2018.

[111] Miran Park, Claude Loverdo, Sebastian J Schreiber, and James O Lloyd-Smith. Multiple scales of selection influence the evolutionary emergence of novel pathogens. *Philosophical Transactions of the Royal Society B: Biological Sciences*, 368(1614):20120333, 2013.

[112] Jasmine F Parkinson, Bruno Gobin, and William OH Hughes. The more, the merrier? Obligate symbiont density changes over time under controlled environmental conditions, yet holds no clear fitness consequences. *Physiological Entomology*, 42(2):163–172, 2017.

[113] Steven R Parratt and Anna-Liisa Laine. Pathogen dynamics under both bottom-up host resistance and top-down hyperparasite attack. *Journal of Applied Ecology*, 55:2976–2985, 2018.

[114] KM Pepin, I Volkov, JR Banavar, CO Wilke, and BT Grenfell. Phenotypic differences in viral immune escape explained by linking within-host dynamics to host-population immunity. *Journal of Theoretical Biology*, 265(4):501–510, 2010.

[115] Luis Ignacio Perez, Pedro Emilio Gundel, Claudio Marco Ghersa, and Marina Omacini. Family issues: fungal endophyte protects host grass from the closely related pathogen *Claviceps purpurea*. *Fungal Ecology*, 6(5):379–386, 2013.

[116] Sarah E Perkins and Andy Fenton. Helminths as vectors of pathogens in vertebrate hosts: A theoretical approach. *International Journal for Parasitology*, 36(8):887–894, 2006.

[117] Joe Pharaon and Chris Bauch. The influence of social behavior on competition between virulent pathogen strains. *Journal of Theoretical Biology*, 455:47–53, 2018.

[118] Romain Pigeault, Julien Vézilier, Stéphane Cornet, Flore Zélé, Antoine Nicot, Philippe Perret, Sylvain Gandon, and Ana Rivero. Avian malaria: a new lease of life for an old experimental model to study the evolutionary ecology of *Plasmodium*. *Philosophical Transactions of the Royal Society of London B: Biological Sciences*, 370(1675):20140300, 2015.

[119] Shai Pilosof, Gili Greenbaum, Boris R Krasnov, and Yuval R Zelnik. Asymmetric disease dynamics in multihost interconnected networks. *Journal of Theoretical Biology*, 430:237–244, 2017.

[120] Ville Pimenoff, Charlotte Houldcroft, Riaan Rifkin, and Simon Underdown. The role of aDNA in understanding the coevolutionary patterns of human sexually transmitted infections. *Genes*, 9:317, 2018.

[121] Helen Piontkivska, Luis F Matos, Sinu Paul, Brian Scharfenberg, William G Farmerie, Michael M Miyamoto, and Marta L Wayne. Role of host-driven mutagenesis in determining genome evolution of sigma virus (DMelSV; *Rhabdoviridae*) in *Drosophila melanogaster*. *Genome Biology and Evolution*, 8(9):2952–2963, 2016.

[122] Laura C Pollitt, Thomas S Churcher, Emma J Dawes, Shahid M Khan, Mohammed Sajid, María-Gloria Basáñez, Nick Colegrave, and Sarah E Reece. Costs of crowding for the transmission of malaria parasites. *Evolutionary Applications*, 6:617–629, 2013.

[123] Laura C Pollitt, Paula MacGregor, Keith Matthews, and Sarah E Reece. Malaria and trypanosome transmission: different parasites, same rules? *Trends in Parasitology*, 27(5):197–203, 2011.

[124] Laura C Pollitt, Nicole Mideo, Damien R Drew, Petra Schneider, Nick Colegrave, and Sarah E Reece. Competition and the evolution of reproductive restraint in malaria parasites. *The American Naturalist*, 177(3):358–367, 2011.

[125] Z Rapti and CE Cáceres. Effects of intrinsic and extrinsic host mortality on disease spread. *Bulletin of Mathematical Biology*, 78(2):235–253, 2016.

[126] Jonathan M Read and Matt J Keeling. Disease evolution across a range of spatio-temporal scales. *Theoretical Population Biology*, 70(2):201–213, 2006.

[127] Karsten Reckardt and Gerald Kerth. Does the mode of transmission between hosts affect the host choice strategies of parasites? Implications from a field study on bat fly and wing mite infestation of Bechstein's bats. *Oikos*, 118(2):183–190, 2009.

[128] Sarah E Reece, Kimberley F Prior, and Nicole Mideo. The life and times of parasites: rhythms in strategies for within-host survival and between-host transmission. *Journal of Biological Rhythms*, 32(6):516–533, 2017.

[129] Roland R Regoes, Steven Hamblin, and Mark M Tanaka. Viral mutation rates: modelling the roles of within-host viral dynamics and the trade-off between replication fidelity and speed. *Proceedings of the Royal Society of London B: Biological Sciences*, 280(1750):20122047, 2013.

[130] Steven Riley, Christl A Donnelly, and Neil M Ferguson. Robust parameter estimation techniques for stochastic within-host macroparasite models. *Journal of Theoretical Biology*, 225:419–430, 2003.

[131] Claude Rispe and Nancy A Moran. Accumulation of deleterious mutations in endosymbionts: Mullers ratchet with two levels of selection. *The American Naturalist*, 156(4):425–441, 2000.

[132] Olivier Rolin, Sarah J Muse, Chetan Safi, Shokrollah Elahi, Volker Gerdts, Lauren E Hittle, Robert K Ernst, Eric T Harvill, and Andrew Preston. Enzymatic modification of lipid A by ArnT protects *Bordetella bronchiseptica* against cationic peptides and is required for transmission. *Infection and Immunity*, 82(2):491–499, 2014.

[133] Boris Schmid, Can Keşmir, and Rob J de Boer. The specificity and polymorphism of the MHC class I prevents the global adaptation of HIV-1 to the monomorphic proteasome and TAP. *PLoS One*, 3(10):e3525, 2008.

[134] Petra Schneider, Megan A Greischar, Philip LG Birget, Charlotte Repton, Nicole Mideo, and Sarah E Reece. Adaptive plasticity in the gametocyte conversion rate of malaria parasites. *PLoS Pathogens*, 14(11):e1007371, 2018.

[135] Stacy O Scholle, Rolf JF Ypma, Alun L Lloyd, and Katia Koelle. Viral substitution rate variation can arise from the interplay between within-host and epidemiological dynamics. *The American Naturalist*, 182(4):494–513, 2013.

[136] Elizabeth SC Scordato and Melissa R Kardish. Prevalence and beta diversity in avian malaria communities: host species is a better predictor than geography. *Journal of Animal Ecology*, 83(6):1387–1397, 2014.

[137] Maite Severins. A modeller's perspective on infection dynamics within and between hosts. *Veterinary Quarterly*, 32(3-4):169–178, 2012.

[138] Maite Severins, Don Klinkenberg, and Hans Heesterbeek. How selection forces dictate the variant surface antigens used by malaria parasites. *Journal of The Royal Society Interface*, page rsif20110239, 2011.

[139] Mingwang Shen, Yanni Xiao, and Libin Rong. Global stability of an infection-age structured HIV-1 model linking within-host and between-host dynamics. *Mathematical Biosciences*, 263:37–50, 2015.

[140] Sourya Shrestha, Ottar N Bjørnstad, and Aaron A King. Evolution of acuteness in pathogen metapopulations: conflicts between classical and invasion-persistence trade-offs. *Theoretical Ecology*, 7(3):299–311, 2014.

[141] David RM Smith and Nicole Mideo. Modelling the evolution of HIV-1 virulence in response to imperfect therapy and prophylaxis. *Evolutionary Applications*, 10(3):297–309, 2017.

[142] Jeff Smith. The social evolution of bacterial pathogenesis. *Proceedings of the Royal Society of London B: Biological Sciences*, 268(1462):61–69, 2001.

[143] Mircea T Sofonea, Samuel Alizon, and Yannis Michalakis. Exposing the diversity of multiple infection patterns. *Journal of Theoretical Biology*, 419:278–289, 2017.

[144] Shelby H Steinmeyer, Claus O Wilke, and Kim M Pepin. Methods of modelling viral disease dynamics across the within-and between-host scales: the impact of virus dose on host population immunity. *Philosophical Transactions of the Royal Society of London B: Biological Sciences*, 365(1548):1931–1941, 2010.

[145] Eleanore D Sternberg, Thierry Lefèvre, Amanda H Rawstern, and Jacobus C de Roode. A virulent parasite can provide protection against a lethal parasitoid. *Infection, Genetics and Evolution*, 11(2):399–406, 2011.

[146] Xiaodan Sun and Yanni Xiao. Multiscale system for environmentally-driven infectious disease with threshold control strategy. *International Journal of Bifurcation and Chaos*, 28(05):1850064, 2018.

[147] Xiaodan Sun, Yanni Xiao, Sanyi Tang, Zhihang Peng, Jianhong Wu, and Ning Wang. Early HAART initiation may not reduce actual reproduction number and prevalence of MSM infection: perspectives from coupled within-and between-host modelling studies of Chinese MSM populations. *PLoS One*, 11(3):e0150513, 2016.

[148] Hanna Susi, Benoit Barrès, Pedro F Vale, and Anna-Liisa Laine. Co-infection alters population dynamics of infectious disease. *Nature Communications*, 6:5975, 2015.

[149] Hanna Susi, Pedro F Vale, and Anna-Liisa Laine. Host genotype and coinfection modify the relationship of within and between host transmission. *The American Naturalist*, 186(2):252–263, 2015.

[150] Maria Svensson-Coelho and Robert E Ricklefs. Host phylogeography and beta diversity in avian haemosporidian (Plasmodiidae) assemblages of the Lesser Antilles. *Journal of Animal Ecology*, 80(5):938–946, 2011.

[151] LH Taylor, F Katzer, BR Shiels, and SC Welburn. Genetic and phenotypic analysis of Tunisian *Theileria annulata* clones. *Parasitology*, 126:241–252, 2003.

[152] Kristof Theys, Pieter Libin, Andrea-Clemencia Pineda-Pena, Ann Nowe, Anne-Mieke Vandamme, and Ana B Abecasis. The impact of HIV-1 within-host evolution on transmission dynamics. *Current Opinion in Virology*, 28:92–101, 2018.

[153] Y Ulrich, BM Sadd, and P Schmid-Hempel. Strain filtering and transmission of a mixed infection in a social insect. *Journal of Evolutionary Biology*, 24(2):354–362, 2011.

[154] Sander P van Noort, Marta C Nunes, Gareth D Weedall, Lars Hviid, and M Gabriela M Gomes. Immune selection and within-host competition can structure the repertoire of variant surface antigens in *Plasmodium falciparum*-a mathematical model. *PLoS One*, 5(3):e9778, 2010.

[155] Kimberly L VanderWaal and Vanessa O Ezenwa. Heterogeneity in pathogen transmission: mechanisms and methodology. *Functional Ecology*, 30(10):1606–1622, 2016.

[156] Fabrice Vavre, Pierre Fouillet, and Frédéric Fleury. Between-and within-host species selection on cytoplasmic incompatibility–inducing Wolbachia in haplodiploids. *Evolution*, 57(2):421–427, 2003.

[157] M Verenini. Parasitoid development within hosts infected by microorganisms. *Bollettino dell'Istituto di Entomologia della Universita degli Studi di Bologna*, 37:225–265, 1983.

[158] LM Viljoen, L Hemerik, and J Molenaar. R_0: Host longevity matters. *Acta Biotheoretica*, 66:1–16, 2018.

[159] Bram Vrancken, Andrew Rambaut, Guy Baele, Anne-Mieke Vandamme, Kristel Van Laethem, Eric Van Wijngaerden, Alexei Drummond, Marc Suchard, and Philippe Lemey. Reconstruction of an HIV transmission chain in a Bayesian coalescent framework. In *Proceedings IWBBIO 2013*, pages 555–556, 2013.

[160] Bram Vrancken, Marc A Suchard, and Philippe Lemey. Accurate quantification of within-and between-host HBV evolutionary rates requires explicit transmission chain modelling. *Virus Evolution*, 3(2), 2017.

[161] Josephine G Walker, Michaela Plein, Eric R Morgan, and Peter A Vesk. Uncertain links in host-parasite networks: lessons for parasite transmission in a multi-host system. *Philosophical Transaction of the Royal Society B: Biological Sciences*, 372(1719):20160095, 2017.

[162] Timothy M Walker, Camilla LC Ip, Ruth H Harrell, Jason T Evans, Georgia Kapatai, Martin J Dedicoat, David W Eyre, Daniel J Wilson, Peter M Hawkey, Derrick W Crook, et al. Whole-genome sequencing to delineate Mycobacterium tuberculosis outbreaks: a retrospective observational study. *The Lancet Infectious Diseases*, 13(2):137–146, 2013.

[163] Xueying Wang and Jin Wang. Disease dynamics in a coupled cholera model linking within-host and between-host interactions. *Journal of Biological Dynamics*, 11(sup1):238–262, 2017.

[164] Brian R Wasik, Ambika Bhushan, C Brandon Ogbunugafor, and Paul E Turner. Delayed transmission selects for increased survival of vesicular stomatitis virus. *Evolution*, 69(1):117–125, 2015.

[165] Sam Wilks, Miranda de Graaf, Derek J Smith, and David F Burke. A review of influenza haemagglutinin receptor binding as it relates to pandemic properties. *Vaccine*, 30(29):4369–4376, 2012.

[166] Lander Willem, Frederik Verelst, Joke Bilcke, Niel Hens, and Philippe Beutels. Lessons from a decade of individual-based models for infectious disease transmission: a systematic review (2006-2015). *BMC Infectious Diseases*, 17(1):612, 2017.

[167] Anouk Willemsen, Mark P Zwart, and Santiago F Elena. High virulence does not necessarily impede viral adaptation to a new host: a case study using a plant RNA virus. *BMC Evolutionary Biology*, 17(1):25, 2017.

[168] Chris Wymant, Matthew Hall, Oliver Ratmann, David Bonsall, Tanya Golubchik, Mariateresa de Cesare, Astrid Gall, Marion Cornelissen, Christophe Fraser, The Maela Pneumococcal Collaboration STOP-HCV Consortium, and The BEEHIVE Collaboration. PHYLOSCANNER: inferring transmission from within-and between-host pathogen genetic diversity. *Molecular Biology and Evolution*, 35(3):719–733, 2017.

[169] Yanyu Xiao and Xingfu Zou. Can multiple malaria species co-persist? *SIAM Journal on Applied Mathematics*, 73(1):351–373, 2013.

[170] Chayu Yang, Drew Posny, Feng Bao, and Jin Wang. A multi-scale cholera model linking between-host and within-host dynamics. *International Journal of Biomathematics*, 11(03):1850034, 2018.

[171] Qinglong Zeng and Allen Rodrigo. Neutral models of short-term microbiome dynamics with host subpopulation structure and migration limitation. *Microbiome*, 6:80, 2018.

[172] Roman Zug, Arnulf Koehncke, and Peter Hammerstein. Epidemiology in evolutionary time: the case of Wolbachia horizontal transmission between arthropod host species. *Journal of Evolutionary Biology*, 25(11):2149–2160, 2012.

[173] JL Zung, S Lewengrub, MA Rudzinska, A Spielman, SR Telford, and J Piesman. Fine structural evidence for the penetration of the Lyme disease *spirochete Borrelia burgdorferi* through the gut and salivary tissues of *Ixodes dammini*. *Canadian Journal of Zoology*, 67(7):1737–1748, 1989.

[174] Mark P Zwart, Wopke Van Der Werf, Monique M Van Oers, Lia Hemerik, Jan Van Lent, JAGM De Visser, Just M Vlak, and Jenny S Cory. Mixed infections and the competitive fitness of faster-acting genetically modified viruses. *Evolutionary Applications*, 2(2):209–221, 2009.
