# Supplementary figures and images for: Linked within-host and between-host models and data for infectious diseases: a systematic review
Source: PeerJ. 2019 Jun 19;7:e7057. doi: 10.7717/peerj.7057 (PMC6589080; doi:10.7717/peerj.7057)

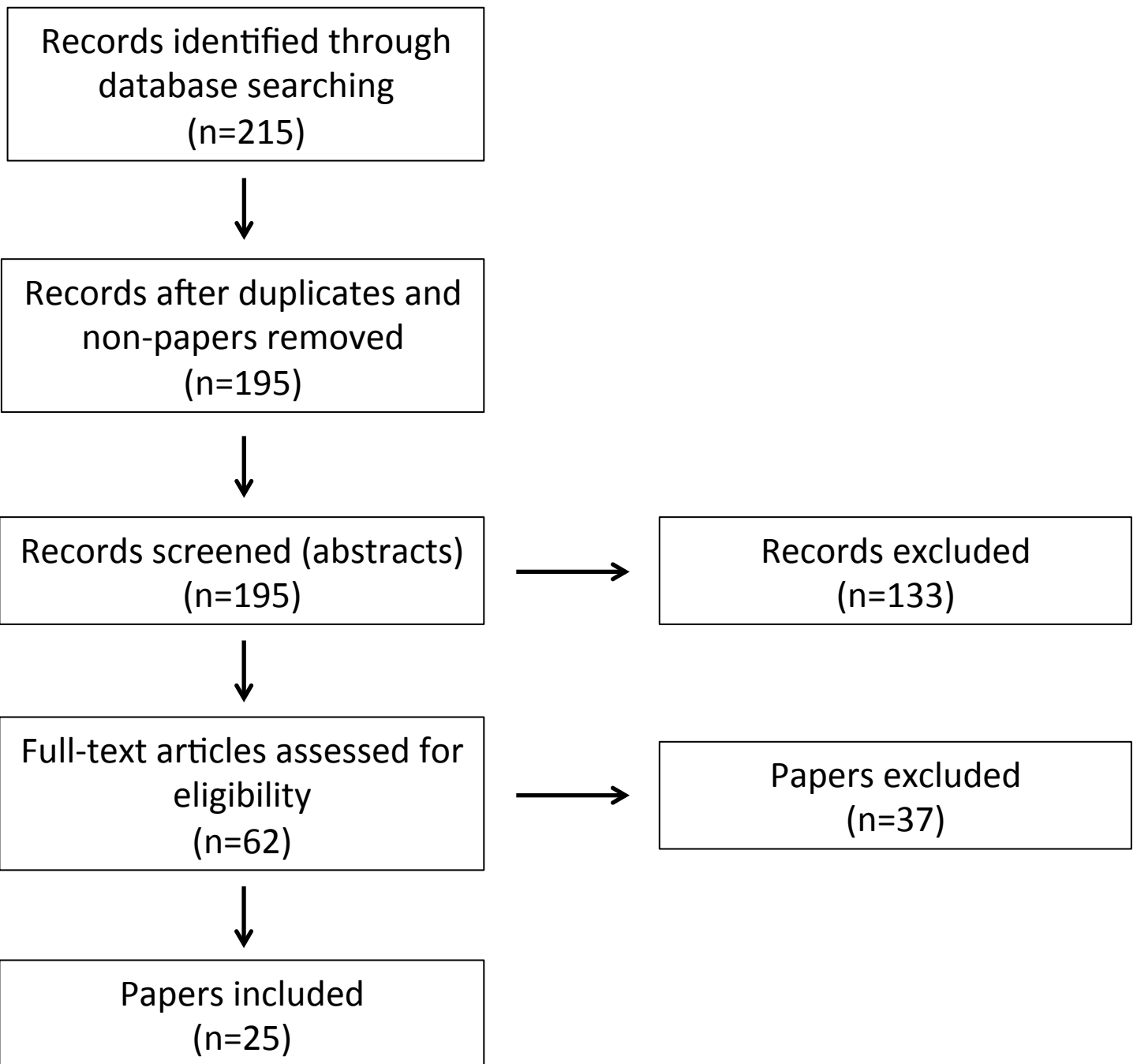

Supplement: Supplemental Information 9 [file peerj-07-7057-s009.pdf]
