# Supplementary material for: Linked within-host and between-host models and data for infectious diseases: a systematic review
Source: PeerJ. 2019 Jun 19;7:e7057. doi: 10.7717/peerj.7057 (PMC6589080; doi:10.7717/peerj.7057)
Supplement: Supplemental Information 10 [file peerj-07-7057-s010.pdf]

Multi-scale models and data for infectious diseases: A systematic review

Rationale and contribution for systematic review

The rationale for conducting this systematic review on multi-scale models for infectious disease incorporating data is to determine the current state of the field and to identify gaps in the incorporation of data. Previously published reports, primarily not systematic reviews, noted the lack of data integrated in multi-scale models, but did not delve into specifics of which scales and biological applications were under-represented.

Our systematic review gives an overview of the papers published on multi-scale models for infectious disease incorporating data, as of the end of November 2017, and identifies areas where more research is warranted. From our original identification of 139 papers, a mere 19 papers satisfied our criteria of models for infectious disease with two scales of dynamics integrating data at least one scale or in the link between them. We extensively analyzed these papers to determine which types of modeling, linking and data were used. Furthermore, we evaluated all the excluded papers to understand what aspect of our criteria was lacking, giving insight to work in the broader field.
